# The Pathogenesis of *Staphylococcus aureus* Eye Infections

**DOI:** 10.3390/pathogens7010009

**Published:** 2018-01-10

**Authors:** Richard J. O’Callaghan

**Affiliations:** Department of Microbiology and Immunology, University of Mississippi Medical Center, 2500 N. State St., Jackson, MS 39216, USA; rocallaghan@umc.edu

**Keywords:** dacryocystitis, blepharitis, conjunctivitis, keratitis, endophthalmitis, ocular host defenses, virulence factors, ocular host response

## Abstract

*Staphylococcus aureus* is a major pathogen of the eye able to infect the tear duct, eyelid, conjunctiva, cornea, anterior and posterior chambers, and the vitreous chamber. Of these infections, those involving the cornea (keratitis) or the inner chambers of the eye (endophthalmitis) are the most threatening because of their potential to cause a loss in visual acuity or even blindness. Each of these ocular sites is protected by the constitutive expression of a variety of antimicrobial factors and these defenses are augmented by a protective host response to the organism. Such infections often involve a predisposing factor that weakens the defenses, such as the use of contact lenses prior to the development of bacterial keratitis or, for endophthalmitis, the trauma caused by cataract surgery or intravitreal injection. The structural carbohydrates of the bacterial surface induce an inflammatory response able to reduce the bacterial load, but contribute to the tissue damage. A variety of bacterial secreted proteins including alpha-toxin, beta-toxin, gamma-toxin, Panton-Valentine leukocidin and other two-component leukocidins mediate tissue damage and contribute to the induction of the inflammatory response. Quantitative animal models of keratitis and endophthalmitis have provided insights into the *S. aureus* virulence and host factors active in limiting such infections.

## 1. Introduction

In addition to causing skin and soft tissue infections, osteomyelitis, endocarditis, blood-borne infections, and pneumonia, *Staphylococcus aureus* is among the most common causes of ocular infections, including blepharitis, dacryocystitis, conjunctivitis, keratitis, and endophthalmitis [1,2,3,4,5,6,7,8]. Approximately 35% of the general public and 50–66% of hospital workers become colonized with the organism [9,10]. Interactions between *S. aureus* and other bacteria of the nasal flora appear to aid or retard the growth of *S. aureus* in the anterior nares [11]. Humans are not the only reservoir for this organism because the organism can be isolated from companion animals, livestock, and wild animals [12,13,14]. About 4% of dogs and some cats carry *S. aureus* at one or more body sites (e.g., abdomen), including MRSA strains [12]. Additionally, livestock, especially pigs but also chickens and cattle, carry strains of ST398 that have been the cause of human infections [13,15,16]. Animals in wild populations (e.g., chimpanzees) harbor and shed *S. aureus* [14]. Well recognized are human carriers who harbor *S. aureus* in their anterior nares, throat, and perianal body sites; however, the organism is often also found in areas around the human eye [7]. Specific strains found in the flora around the eye provide the organisms that infect the eye; that is, the isolates obtained from eye infections match those found in the patient’s periocular area [17]. The common ocular infections that involve *S. aureus* are listed below starting with those of the peripheral area and moving inward to the vitreous cavity. 

**Figure 1:**
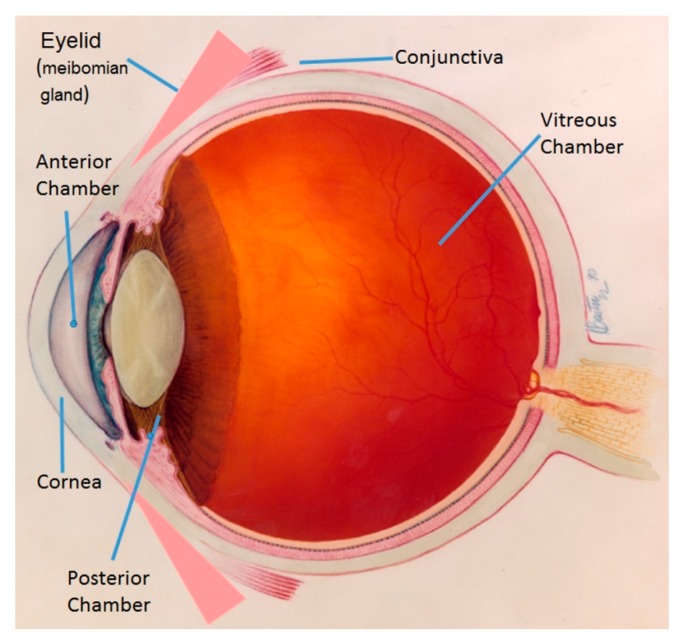
Illustration of the eye. This illustration is a modification of an image on the website of the National Eye Institute of the National Institutes of Health.

A drawing of the human eye is included for orientation (see Figure 1).

## 2. Meibomian Gland and Blepharitis

The eyelid serves to protect the ocular surface and the glands of the eyelid, especially the meibomian gland, provide important secretions for the tear film. Infections of the eyelid (i.e., blepharitis) can cause obstruction of these glands preventing their lipid-rich secretions from contributing to the tear film [18,19]. Included in the tear film is an important top layer of lipid extensively derived from the meibomian gland that limits water evaporation thus helping to prevent the development of a dry eye. Lipases from both the host and bacteria can release free fatty acids, cholesterol, and other lipids that accumulate in the duct contributing to duct blockage and recruitment of neutrophils and other inflammatory components to the gland. Important to these processes are staphylococci that are in the ocular flora and can contribute lipase to the process. An interesting aspect of the infection relates to the stimulation of *S. aureus* growth by cholesterol, a lipid released by the hydrolysis of cholesterol esters [1]. The recommended treatments for these infections are somewhat controversial and range from an oral antibiotic, to solely the application of warm compresses, or even the use of a mechanical device to acquire duct opening [19,20,21]. 

## 3. Dacryocystitis

Excess amounts of tears drain into the nasal sinus through nasolacrimal ducts whose openings (puncta) in the eyelids are adjacent to the nose (not indicated in Figure 1). The nasolacrimal ducts also drain topically applied drugs into the sinuses. Dacryocystitis forms when the nasolacrimal duct is blocked and infection develops due to the overgrowth of organisms in the local flora, most often *S. aureus*, *S. epidermidis*, or pneumococcus [22]. Such an infection often involves the lacrimal gland and can spread to the cornea potentially resulting in corneal ulceration. Patients with dry eye issues often have the duct closed using punctal plugs to limit the drainage of tears down the duct. Dry eye is a compromising condition leading to anterior eye infections so the plugs are useful for retaining tears; however, the plugs can serve as a foundation for the development of a bacterial biofilm [23]. 

The role of *S. epidermidis* as a pathogen has gained acceptance, especially in terms of ocular infections for which there is greater recognition of this organism as a pathogen. Cases of keratitis and endophthalmitis can yield positive cultures of only *S. epidermidis* and these infected eyes undergo damaging inflammation and likely direct damage from bacterial products. Production of exoenzymes and phenol-soluble modulins as well as biofilms have been described as factors active in non-ocular infections by which this organism protects itself from the host response [24]. These same virulence factors can likely explain the pathogenic role of *S. epidermidis* in the eye. 

## 4. Conjunctivitis

A swab rubbed across the conjunctiva of a normal eye will contain the bacteria that comprise the flora of that eye, including coagulase-negative *Staphylococcus*, *Propionibacterium*, *Corynebacterium*, *S. aureus*, *Streptococcus* species, *Micrococcus* species, *Bacillus* species, and *Lactobacillus* species [25]. Germ-free mice are more susceptible to bacterial infection than mice with the normal flora. Creating a population of coagulase-negative staphylococci on the conjunctiva of a formerly germ-free mouse can induce IL-1β, which converts the conjunctiva to a more resistant state as compared to that of germ-free mice [23]. A somewhat different but related finding was obtained when *Corynebacterium mastidis* was allowed to colonize the eye creating a flora that caused an increase in IL-17, which protected the eye from infection [26]. Thus, the studies agree on the importance of the flora to create a cytokine-rich environment that can provide protection against infection. 

Conjunctivitis is a common infection of the ocular surface that is more prevalent in children than adults. Such infections can be transmitted by ocular contact with fingers or fomites or conditions such as dry eye, allergy or virus infection. Viruses are important causes of conjunctivitis and *Chlamydia trachomatis* and *Haemophilus influenzae* are the important bacterial causes of conjunctivitis in children less than one year old [2,4]. *S. epidermidis*, *S. aureus*, and *Streptococcus pneumoniae* are important causes of conjunctivitis in older children. Conjunctivitis can also result as a spread of infection from the eyelid or cornea and can occur as a result of trauma to the eye, including the trauma due to the use of contact lenses [4,27,28].

Conjunctivitis can also frequently be due to ocular allergy without evidence of infection. Such atopic patients do have a significantly greater presence of *S. aureus* on their conjunctiva as compared to non-atopic control patients. The atopic patients experiencing keratoconjunctivitis with corneal ulcers have a significantly greater frequency of both *S. aureus* and the *S. aureus* secreted enterotoxins [29]. The severity of allergic conjunctivitis in mice could be enhanced by the addition of *S. aureus*. *S. aureus* was reported to react with Toll-like receptor 2 resulting in increased proinflammatory cytokines and an increased quantity of IgE antibody in patient sera [30]. 

The conjunctiva does possess a host defense capability that is mediated by conjunctival goblet cells [31]. The goblet cells, when exposed to toxin producing *S. aureus*, respond by activating the caspase 1 pathway. Activation of the caspase 1 pathway results in the production of IL-1β, a potent inducer of inflammation. Because of the host defenses of the conjunctiva, injection of any of four strains of *S. aureus* (10^5^ Colony Forming Units [CFU]) into the rabbit conjunctival sac failed to produce significant conjunctivitis or growth of the bacteria [32]. One strain (UMCR1) injected into the conjunctival sac was found to achieve bacterial growth (i.e., increased CFU over the number injected) and to produce a clinical score significantly higher than the other strains tested. Bacterial virulence factors mediating conjunctivitis are unknown, but a subsequent study showed that UMCR1 invaded human corneal epithelial cells in vitro with 10-fold greater efficiency [33]. Virulence factors facilitating the unique capabilities of UMCR1 are not known despite analysis of its DNA genome. 

## 5. Keratitis

### 5.1. Introduction

The infection of the cornea is a sight-threatening infection because the cornea must be strong enough to maintain the integrity of the eye yet clear enough to allow the passage of light. Infections of the cornea can result in bacterial and host response reactions that damage the corneal structure possibly leading to its rupture with devastating consequences to the eye or to a healing process that results in a scar that blocks the passage of light. Corneal transplants are often needed to restore vision, but transplants are challenging in an eye that has undergone a serious corneal infection. 

Despite the great availability of organisms associated with the skin surrounding the eye, keratitis infrequently develops. This is true because the eye provides a diverse collection of antimicrobial factors, especially in the tear film, that protect the cornea from infection (Table 1). The topical application of *S. aureus* in large numbers (e.g., 10^7^ CFU) to a scarified rabbit cornea typically fails to cause an infection and results in the rapid loss of the bacteria [34]. Tears have three layers; namely, the lipid-rich or meibomian layer that covers the surface of the tears limiting their evaporation, the aqueous layer that lubricates the anterior eye and provides multiple antibacterial proteins and peptides, and a mucous layer that interacts with the corneal epithelial cells [35]. The meibomian glands secrete lipids and this activity is controlled by hormones (androgens) and possibly by nerves. The lipid layer contains non-polar sterol esters and wax whereas the polar portion of the lipid layer contains phospholipids. The aqueous layer of the tear film is derived from the lacrimal gland and contains sIgA, IgG, and IgM immunoglobulins. The IgA molecules are more prevalent than the other immunoglobulins and those that recognize bacterial adhesins can prevent bacterial attachment to cells of the corneal or conjunctival epithelium.

In addition to antibacterial proteins in the aqueous layer, there are antimicrobial peptides (AMP) that can bind to and kill bacteria. The antimicrobial peptides with well recognized activity are cathelicidin LL-37 and beta-defensins [36,37,38]. LL-37 has considerable bactericidal activity initiated by binding to the surface of multiple types of bacteria, including *S. aureus* [39]. The beta-defensins 1-6 have been reported to be present in the aqueous layer, but there have been concerns that their in vitro actions on bacteria could be retarded by unknown factors in the tears [40,41]. The interaction of *S. aureus* with the epithelial cells results in the production of multiple cytokines [42,43,44,45] and the pro-inflammatory cytokines induce an infiltration of neutrophils and the production of the chemokine CCL20, a chemotactic factor especially for dendritic cells and an anti-bacterial factor [38]. 

*S. aureus* produces numerous virulence factors and a key function of multiple virulence factors is to protect the organism from specific components of the host defense. The ability of surfactant D in tears to promote clearance of *S. aureus* was demonstrated by comparing the susceptibility to corneal infection of wild type mice to surfactant D knockout mice [52]. The wild type mice infected with *S. aureus* experienced less ocular injury as compared to the knockout mice, indicating the protective role of surfactant D. Furthermore, the protease inhibitor E_64_ protected surfactant D from a secreted bacterial cysteine protease allowing surfactant D to remain active in subjecting bacteria to phagocytosis [50]. These findings illustrate the role of the bacterial protease in infection and imply that inhibitors of bacterial proteases could render the invading bacteria more susceptible to host defense systems. 

Other components of protection against infection that are active in the aqueous portion of the tear film are phospholipase A2 and antimicrobial molecules whose activities can increase in response of the epithelial cells to the presence of the bacteria. Once infection occurs in the anterior portion of the eye, the amount of phospholipase A2 increased by five-fold thus enhancing protection against bacterial infection [55]. Another responsive factor is the production of keratins whose cleavage creates antimicrobial peptides called KDAMP’s. These peptides can kill a variety of bacteria including both *S. aureus* and *S. epidermidis* [56]. Also, human corneal epithelial cells can produce beta-defensin 2 (hBD-2), a potent antimicrobial factor [57]. Bacteria and bacterial products have been found to induce the expression and release of hBD-2 and this molecule has a lethal effect on *S. aureus* and other bacteria [58].

The mucous layer of the tear film is in contact with the corneal and conjunctival epithelium forming the last barrier against bacterial interaction with these tissues. The mucous layer contains several forms of mucin, immunoglobulins, and leukocytes [35]. The mucins form the major component of this layer and are derived from the lacrimal gland, conjunctival goblet cells, and corneal and conjunctival epithelial cells. Seven different mucin molecules are known to be in or in contact with the mucous layer; namely, mucins 2, 5AC, and 7 are found in a soluble form, whereas mucin 4 and 5B can be membrane bound, and mucin 1 and 16 are membrane bound. Mucins, particularly mucin 16, extend from the surface of epithelial cells of the cornea and conjunctiva and form a thick barrier that limits the binding of *S. aureus* to the epithelial cells [38,59,60,61]. The mucin barrier also limits the need for the induction of inflammatory reactions involving these tissues. Glycans are also found on the surface of the corneal epithelial cells and they also serve as an obstacle to bacterial attachment to the cell surface [33].

The survival of bacteria in the tear film can result in the infection of the cornea, an event that is favored by any of a variety of predisposing factors, including dry eye, corneal trauma (abrasion), ocular allergy, viral infection, ocular surgery, and contact lens usage [5,62,63]. Unlike the infections of the eyelid or conjunctiva, keratitis has the potential to cause a severe loss in visual acuity and even blindness. The mechanisms involved in the initiation of keratitis are not well understood, but it is known that *S. aureus* can bind to human corneal cells, a reaction mediated by the fibronectin-binding protein on the bacterial surface [64]. In addition to fibronectin-mediated binding, tissue binding of *S. aureus* can also be mediated by the collagen-binding adhesin. Adhesion of *S. aureus* to the rabbit cornea in vivo for a parent strain having the collagen-binding adhesin was found to be more efficient in causing keratitis than its isogenic mutant lacking the collagen-binding adhesin [65]. The binding of the wild type strain via its collagen-binding adhesin could be inhibited by adding a recombinant collagen peptide that competed with the binding of the bacteria to the cell. Adhesion can be followed by internalization of the bacteria and differences in the efficiency of internalization exist among *S. aureus* strains and could possibly correlate with virulence [33]. Alternatively, the process of internalization could help protect the cornea because the outer epithelial cells can be shed removing some of the internalized bacteria from the cornea [36]. 

Passage of the bacteria into and through the epithelial cells does not assure the passage of bacteria into the central cornea (stroma). Under the epithelial cells is a basement membrane that forms a physical barrier rich in collagen type V and laminin with holes too small for bacterial passage [36]. Passage could be achieved by the action of bacterial proteases on this barrier, a mechanism demonstrated in vitro for *Pseudomonas aeruginosa* [66]. *S. aureus* has the ability to produce at least 10 proteases, but production of proteases is repressed by the regulator known as repressor of toxin (Rot) and is up-regulated by the accessory gene regulator (Agr) system [67]. Since Rot is active early in the growth cycle prior to the Agr activation in late log phase, production of the well-recognized proteases could require extensive bacterial replication before achieving protease production. However, Rot does up-regulate the superantigen-like proteins (SSLs) [68,69] and preliminary findings in this laboratory indicate that at least one SSL contributes to virulence during *S. aureus* keratitis (unpublished finding). Since SSLs are produced early in the growth cycle, SSLs could aid in initiating keratitis. 

Secretory phospholipase A2 (PLA2) was described as mediating a substantial defense in tears against gram-positive bacteria, with nanogram quantities being able to kill *S. aureus* in vitro [49]. The amount of PLA2 activity in the rabbit tear film decreased by >70% with increased age from 10 weeks to 28 weeks of age, however, there was considerable PLA2 activity even at 28 weeks as demonstrated by the PLA2-mediated cleavage of fatty acid from the *S. aureus* surface [55]. This amount of host defense against *S. aureus* could explain why individuals do not commonly suffer corneal infections and why predisposing factors (e.g., dry eye) favor the development of an infection. The killing of *S. aureus* by rabbit tears was inhibited in vitro by spermidine [34]. The importance of PLA2 to the protection of the rabbit eye from infection by *S. aureus* was demonstrated by the ability of *S. aureus* on contact lenses to infect scarified rabbit corneas provided the eyes were treated with spermidine [70]. The administration of viable *S. aureus* to the tear film or to the surface of the cornea, even after scarification, typically results in the rapid clearance of the bacteria [33]. One strain (UMCR1) of *S. aureus*, which is susceptible to rabbit tears in vitro, was able to topically infect a scarified cornea without use of spermidine [33]. This is the same unusual strain that could replicate in the rabbit conjunctival sac causing conjunctivitis [32]. The mechanism(s) by which UMCR1 achieves the infections of the conjunctiva and cornea is not known, but could relate to its greater ability to penetrate corneal epithelial cells in vitro [33]. Interestingly, the mouse cornea, unlike the rabbit cornea, is readily infected by applying a topical drop of a typical *S. aureus* strain to a scarified cornea [71]. The possibility exists that keratitis is dependent on rapid bacterial penetration of the corneal epithelium.

### 5.2. Bacterial Virulence

Numerous studies of keratitis in rabbits have employed a simpler model in which the bacteria are injected directly into the rabbit central cornea (i.e., corneal stroma). In the rabbit corneal injection model, log phase *S. aureus* (10^2^ CFU in 10 µL of tryptic soy broth) injected into the cornea (stroma) replicate to 10^3^ CFU by 5 h postinfection (PI), 10^7^ CFU by 16 h PI, and maintain over 10^7^ CFU through 24 h of infection [55]. Injection of log phase bacteria into the corneal stroma results in an unusually precise animal model of infection featuring both reproducible bacterial growth and pathological changes. The infection with wild type *S. aureus* results in severe inflammatory changes of the cornea (edema, neutrophil infiltration), sloughing of the corneal epithelial cells, fibrin accumulation in the anterior chamber and severe iritis, including blanching of the iris (loss of blood flow) [72,73]. Infection of the cornea also causes intense inflammation of the eyelid with neutrophils migrating from eyelid vessels to the tear film [74]. In both rabbits and mice, the age of the infected animal determined the extent of the inflammation; more extensive inflammation occurred in young rabbits than aged rabbits, and more inflammation occurred in aged mice than in young mice [75,76].

The virulence of a prototype *S. aureus* strain (8325-4) injected into the rabbit cornea was significantly reduced by a mutation of the gene coding for alpha-toxin [72,73]. The mutant strain supplemented with a functional alpha-toxin gene (rescue strain) had corneal virulence equivalent to that of the parent strain. The growth of the parent, mutant and rescue strains was not significantly different in the cornea. Similar findings illustrating the importance of alpha-toxin were subsequently obtained for strain Newman [77]. Nanogram quantities of purified alpha-toxin were shown to cause extensive sloughing of the corneal epithelium, corneal edema, and severe inflammation and blanching of the iris [73,78]. The central role of alpha-toxin in corneal damage was found also in the infection of the mouse cornea [71,79]. Additionally, alpha-toxin has been linked to the ulcers that form in the human peripheral cornea during the use of contact lenses [80]. There is a possibility that corneal ulcer formation during *S. aureus* keratitis involves both alpha-toxin and *S. aureus* elastase [81]. Severe ulcers in rabbits with contact lenses were observed only when alpha-toxin was produced. These findings for the cornea are compatible with the role that alpha-toxin is considered to have during *S. aureus* pneumonia in humans [82]. 

Research on alpha-toxin’s mechanism of action indicates that the toxin binds to the metalloproteinase ADAM 10 and enters the cytoplasmic membrane before moving laterally until seven subunits unite into a circular arrangement forming a pore through the cytoplasmic membrane [83,84]. The pore allows small molecules to exit the cell and calcium to enter causing a cellular dysregulation. Alpha-toxin lyses neutrophils, platelets, monocytes, T cells, pneumocytes, keratinocytes, and endothelial cells [83]. Alpha-toxin binding activates its receptor protease, ADAM 10, which then cleaves the E-cadherin molecules involved in attaching cells to a membrane, as happens to the corneal epithelium when subjected to alpha-toxin [70,75]. The production and secretion of active alpha-toxin is maximally regulated by the Agr regulator system that triggers a 50-fold increase in alpha-toxin production starting late in the log phase [85,86]. Other regulators also affect the production of alpha-toxin, including Sar, Sae and SarH1 such that the toxin production depends on the interaction of multiple regulators [87,88]. In the early phase of growth, before the Agr system is activated, alpha-toxin production has been linked to the production of phenol-soluble-modulin peptides, which are important to virulence [89]. 

Because of the potency of alpha-toxin in the cornea, efforts have been made to inhibit its action on the cornea. Combinations of lipid with cyclodextrin have been shown to inhibit alpha-toxin mediated lysis of erythrocytes and one such inhibitor containing cholesterol has been shown to reduce the severity of *S. aureus* keratitis in the rabbit eye [90,91]. Antibody to alpha-toxin neutralizes the toxin in vitro and prior immunization with the alpha-toxin toxoid protects the rabbit cornea from the severe effects of the toxin during keratitis [77,92]. An alpha-toxin specific monoclonal antibody applied topically to infected rabbit corneas was unable to provide significant corneal protection, but the Fab fragment of this monoclonal antibody applied topically was effective in limiting the corneal epithelial erosion appearing during *S. aureus* keratitis [93]. Apparently, the smaller size of the Fab fragment allowed better corneal penetration than that obtained by the intact antibody molecule. 

The study of *S. aureus* mutants with knockouts in their beta-toxin or protein A gene showed that these two genes do not contribute significantly to corneal virulence; that is, the growth and pathological effects of infection with the mutant and parent strain were not significantly different [72,73,77]. The beta-toxin knockout strain failed to produce scleral edema that occurred in eyes infected in the cornea with the wild type strain. Injections of purified beta-toxin into rabbit corneas caused substantial amounts of scleral edema [73]. The extensive action of beta-toxin, a sphingomyelinase, on the sclera and not the cornea, reflects the high sphingomyelin content of the scleral epithelium and the relatively low sphingomyelin content of the cornea. The strain deficient in protein A produced corneal virulence, as measured by the clinical score, that was equivalent to the wild type strain producing protein A [72]. 

In contrast to beta-toxin, both gamma-toxin and Panton-Valentine leukocidin (PVL) were found to significantly contribute to corneal virulence [77,94]. Gamma-toxin is composed of an F component and an S component that are each non-toxic when tested alone [95,96]. The S component binds to the target cell and only then will the F component bind [96]. The combination of F and S can move laterally in the cell membrane and multiple F-S pairs can combine into a ring that penetrates the membrane causing cell lysis [96,97,98]. The infection of rabbit corneas with a gamma-toxin deficient mutant produced a clinical score that was significantly lower than that of the parent and the rescue strains [77]. The injection of gamma-toxin into the cornea caused neutrophil infiltration of the cornea and iris as well as conjunctival reddening (injection) and chemosis (edema), and fibrin accumulation in the anterior chamber [99]. Antibody to the native form of gamma-toxin, but not to the recombinant form, protected the eye from toxin activity [99]. 

PVL is another two-component toxin composed of an F and an S protein that is produced mainly by community-associated MRSA strains (CA-MRSA), which are frequently found as the cause of skin and soft-tissue infections and are recognized as important agents of keratitis [100,101,102,103]. One study has determined that PVL producing strains likely cause a more severe form of keratitis, a form that requires surgery more often than keratitis caused by non-PVL producing strains [104]. Corneal infection of mice with three parent strains or three PVL knockout strains were compared [94]. In an USA 300 strain, the loss of the functional PVL gene caused a significant reduction in virulence whereas the loss of the functional PVL gene in the USA400 strains resulted in less of a reduction in virulence. Over-expression of the PVL gene in USA400 caused an increase in the bacterial burden. Antisera made to PVL components were tested in mice to determine if they provided protection, however, the sera employed were not shown to be free of reactivity with other related toxins, especially gamma-toxin and other leucocidin proteins (see below). Thus, PVL appears to contribute to the corneal virulence of the USA300 strains, but the role of PVL in other strains is unconfirmed.

There is a strong relationship among the proteins forming gamma-toxin and those forming PVL and other leukocidins of *S. aureus*. Gamma-toxin is composed of two proteins, HlgB bound to either HlgA or HlgC and likewise PVL is composed of its two proteins, LukF-PV and LukS-PV [96]. Lesser known proteins involved with toxic activity include LukD, an F-type protein, and LukE, an S-type protein [96,97,98]. There is about 60–70% sequence homology among the various F components and a fairly similar degree of homology among the multiple S components [96]. The two-component toxin systems of *S. aureus* are complicated by the fact that the F component of one toxin can bind to the S component of another toxin [96]. Therefore, multiple unique hybrid toxins can be formed by one component from each of two different toxins. Each combination of an F and an S component can have its own specific toxicity. How these hybrid toxins relate to corneal damage is yet to be resolved, but, because these toxins are homologous with gamma-toxin, it is likely that one or more hybrid toxins have corneal toxicity.

In addition to the toxins produced by *S. aureus*, the surface of the organism has molecules important to the infectious process. Both the fibrinogen-binding protein and the collagen-binding adhesin have been described (above) as mediating the binding critical to the initiation of keratitis [65,105]. Also on the bacterial surface is the antigenic carbohydrate designated as poly-*N*-acetylglucosamine (PNAG). This molecule has been targeted with antibody that is able to provide protection against *S. aureus* by limiting both the bacterial load and pathology of the infection [106]. Also active in the infection are lipoproteins that have been shown to react with Toll-like receptor 2 to induce both cytokines (IL-6 and IL-8) and antibacterial molecules. Among the antibacterial molecules induced are hBD-2, LL-37 and iNOS [42].

One of the leading predisposing conditions to keratitis is the use or misuse of contact lenses; in fact, 56% of the corneal ulcers occur in patients who wear contact lenses [107]. Both the lens and lens storage case provide sites for biofilm formation that serve as a reservoir for bacteria. An analysis of bacteria isolated from patients with ocular infections showed that 55% of the *S. aureus* and 34% of the *S. epidermidis* could form a biofilm in a microtiter plate. In contrast to the staphylococci isolates, the *Pseudomonas aeruginosa* isolates from ocular infections failed to form biofilms under the same conditions [108]. Contact lens safety is dependent to a major extent on the cleaning of the lens with a solution able to limit bacteria found on the lens. Three commercial cleaning solutions were found to inhibit biofilm formation by staphylococci, including *S. aureus* [109].

### 5.3. Host Response

The corneal stroma, unlike the epithelial surface and the tear film, lacks significant protection from infection; even non-pathogenic species can replicate efficiently in the corneal stroma [110,111]. The corneal stroma together with the epithelial cells can respond to the presence of bacteria and their products. The normal cornea contains complement able to generate a functional attack complex and the quantity of complement significantly increases when the cornea is challenged with lipopolysaccharide or a ribitol teichoic acid immune complex [112]. Additional corneal responses to infecting organisms include the production of multiple cytokines, including IL-1α, IL-1β, IL-6, IL-8, TNF-α, IL-17, and IL-22 [42,43,44,45,106]. Infection of the rabbit cornea with *S. aureus* induces the expression of the antimicrobial molecule CAP37 by the corneal epithelial cells and stromal fibroblasts, a response intended to limit the bacterial load in the cornea [113]. The response of corneal epithelial cells in vitro to a toxigenic or non-toxigenic strain of *S. aureus* was analyzed in terms of mRNA responses [45]. An interesting finding was that 650 RNA species were affected by the presence of the bacteria, but the difference between the responses to a toxigenic versus a non-toxigenic strain was limited to the induction of only 37 genes by only the toxigenic strain. One finding in this study was the increase in the chemokine CCL20, a molecule that is known to attract lymphocytes (CD4 T cells) and has a direct bactericidal action. 

The importance of cytokines and immune response-related proteins to protection of the cornea has been shown by infection of mice specifically deficient in a functional gene for a specific cytokine or immune-related gene. The IL-6 knockout mice were shown to produce higher bacterial loads and greater neutrophil infiltration than wild type mice infected with *S. aureus* [114]. Knockout mice lacking functional IL-4 also experienced a more severe infection than wild type mice; wild type mice, unlike the knockout mice, were able to up-regulate several cytokines, including IL-6, IL-10, and MIP-2 [43]. Whole killed *S. aureus* or *P. aeruginosa* on contact lens material applied to mouse eyes caused a corneal infiltrate seen as a haze in the cornea [115]. This response to the bacteria could be inhibited by antibody to a component (CD18) of the leukocyte surface protein (LFA-1) normally reactive with ICAM-1. In a similar fashion, lifitegrast, which binds to LFA-1, can also block the corneal infiltration response to the killed bacteria [112]. Mice lacking a functional CXC receptor experienced an infection in which fewer neutrophils infiltrated the cornea allowing the bacterial load to increase relative to that seen in infections of wild type mice [44]. The mice lacking the CXC receptor had high cytokine responses, but there was not an up-regulation of adhesion molecules needed for neutrophil migration to the cornea. Taken together the studies of knockout mice clearly show the importance and diversity of immune-related molecules needed to protect the cornea during a *S. aureus* infection.

### 5.4. Conclusions

The cornea is well protected from surface infections and its immune response is designed to provide an even greater degree of protection. Bacteria injected into the corneal stroma escape the bulk of the immune defenses allowing a variety of secreted toxins to inflict direct tissue damage and to trigger a damaging immune response. 

A summary of major events in *S. aureus* keratitis listed in chronological order (from left to right) is offered in Table 2. 

## 6. Endophthalmitis

### 6.1. Introduction

Endophthalmitis is an infection of the aqueous humor, the vitreous, or both. These inner eye sites are infected by the spread of bacteria from a non-ocular site of infection (endogenous endophthalmitis), from the spread of infection from the cornea, or following local trauma, most commonly surgical trauma or trauma in association with an intra-vitreal injection [116,117]. Infection following cataract surgery is the most common form of endophthalmitis and it is commonly caused by coagulase-negative staphylococci although the more virulent infections involve *S. aureus* [8,118,119]. The incidence of endophthalmitis associated with cataract surgery is less than 0.01% [120]. Because endophthalmitis following surgery is typically caused by ocular surface organisms, the eyelid and surrounding area are treated with povidone iodine, which kills bacteria within seconds [121]. Approximately 60% of the cataract surgeons treat their patients with prophylactic antibiotic, a treatment that has been complicated by the resistance of MRSA strains to multiple antibiotics (especially fourth generation fluoroquinolones) and the problem of a rare but severe allergic reaction affecting the retina (hemorrhagic occlusive retinal vasculitis, HORV) when vancomycin is used [122,123]. Trauma leading to endophthalmitis can also be caused by non-surgical events and these infections are often caused by *Bacillus cereus* or *S. aureus* [124]. Intra-vitreal infections following an intra-vitreal injection are most frequently caused by coagulase-negative staphylococci or *S. aureus*, whereas *S. aureus* and streptococci (especially pneumococci) are the cause of endophthalmitis resulting from the spread of bacteria from an infection in a non-ocular site [8]. 

### 6.2. Bacterial Virulence

Analysis of *S. aureus* strains isolated from endophthalmitis cases at multiple clinical centers showed that five clonal types accounted for 58.9% of the infections [125]. The tissue damage occurring during *S. aureus* infection of the vitreous has been analyzed relative to the bacterial structural or secreted factors responsible for inflammation or direct toxicity. Dead bacteria, bacterial cell walls, and culture supernatants have been compared for their effects on the rabbit vitreous chamber [126]. The dead bacteria or cell walls were found to cause inflammation, but did not significantly damage the retina. However, culture supernatant was toxic for the retina, suggesting that this tissue damage was due to secreted toxin(s), either directly or together with the inflammation they caused [126]. The injection of purified alpha-toxin in its native state or in a heat-inactivated state was analyzed in the rabbit anterior chamber [127]. Alpha-toxin caused extensive inflammation and damage to the iris, but antisera to the toxin or an inhibitor of the toxin action (methyl-β-cyclodextrin-cholesterol) was able to significantly inhibit the toxicity. Injection of *S. aureus* viable organisms, purified protein A, or purified alpha-toxin caused both inflammation and loss of retina function [128]. The role of secreted toxins in damaging the retina was further supported by studies of genetic regulators that control the production of secreted proteins. A comparison of knockout strains lacking a functioning Agr, SarA or both SarA and Agr showed that the knockout lacking Agr or lacking both SarA and Agr had less virulence than the knockout lacking only a functional SarA regulator [129,130]. The knockout strain lacking both SarA and Agr lost about three-fourths of its virulence [130,131]. In addition to the analysis of SarA and Agr, studies of another toxin regulator known as CodY were performed [132]. The repression of virulence genes by CodY is controlled by branched chain amino acids in the environment; the presence of these amino acids permit repression of virulence genes by CodY. When CodY is not binding to the DNA, there is increased expression of metabolic and virulence genes, including beta-toxin, delta-toxin, phenol soluble modulins, toxic shock syndrome toxin 1 (TSST-1), aureolysin protease, and enterotoxin *sec3*. Thus, the amino acid metabolites in the vitreous can help limit the virulence of an infection.

The toxins responsible for tissue damage in the vitreous have been sought by comparing the infections caused by strains with mutations in specific toxin genes to that of their isogenic parent strains and complemented mutants with restored toxin production (rescue strain). Analysis of the gamma-toxin mutant strain relative to its parent and rescue strains showed that gamma-toxin was not needed for significant virulence; that is, the infection with the gamma-toxin deficient mutant was comparable to that of the parent and rescue strains [133]. Other studies of gamma-toxin, based on the injection of toxin protein into the vitreous (see below), show that gamma-toxin is toxic for the vitreous chamber. The findings obtained by the studies of the gamma-toxin mutant and the injection of purified gamma-toxin differ and the reason for this is not clear. Possibly, the toxin injection involved more toxin than is produced during infection by the strain employed in the genetic study. 

Gamma-toxin and PVL are two component leukotoxins of *S. aureus* and the component proteins of these toxins can interact with one another to form hybrid toxins. The injection of gamma-toxin, PVL toxin, or any of four hybrid toxins caused toxicity in the vitreous chamber [134]. The role of these toxins in infections was further confirmed by detecting the expression of PVL and gamma-toxin genes *hlgB* and *hlgC* during the *S. aureus* infection of the vitreous [135]. 

The proteases produced by *S. aureus* have been implicated in the pathogenesis of intravitreal infection relative to the inactivation of a heat-shock protein, beta-crystallin [136]. The bacterial protease responsible for the cleavage of beta-crystallin has not yet been determined. Beta-crystallin prevents apoptosis in the retina and its destruction leads to retinal cell loss. Such apoptosis has been found to be mediated by damage to the mitochondria that can release an apoptosis inducing factor [137]. 

### 6.3. Host Response to Infection

An important feature of bacteria that mediate infections starting in the anterior or posterior chamber is their ability to adhere to the intra-ocular lenses used in cataract surgery [138,139,140,141]. The human lens removed at cataract surgery is also able to bind bacteria found at the ocular surface, including *S. aureus* [142]. Damage to the tissue surrounding the lens (capsule) could facilitate the development of endophthalmitis, especially in infections involving bacteria in both the aqueous humor and vitreous humor [143,144]. 

The anterior chamber is recognized as an immune privileged site that remains such despite inflammation that develops in response to bacteria or their products [145]. The aqueous humor is a filtrate of plasma and contains anti-bacterial substances providing considerable protection from infection. The aqueous humor of rabbits can kill a significant number of *S. aureus* within 1 h after injection into the rabbit anterior chamber [146]. Injection of *S. aureus* (10^4^ or 5 × 10^5^ CFU) into the rabbit aqueous humor was found by 24 h to result in culture positive infections in approximately 70% of the infected eyes. The aqueous humor of the culture positive eyes contained only 2.3 or 4.2 log CFU, a number less than that used to infect the eyes [147,148]. One unusual strain (UMCR1) was able to infect all inoculated eyes and to replicate in this site from 10^4^ CFU to almost 10^7^ CFU [149]. Strain UMCR1 caused rapid and severe pathologic effects within 16 h of infection. The lethal action of rabbit aqueous humor for *S. aureus* was found to be mediated by an 8 kDa peptide of unknown identity [150,151]. The aqueous humor has been found to contain 147 different proteins that include host defense molecules such as lipocalin, lysozyme, complement, and immunoglobulin [152]. Also present in the aqueous humor is at least one defensin (hBD-1) [41,153,154]. The activation of complement in the aqueous includes the presence of C3, C4a, and C5a that provide potent chemotactic activities. In addition to these constitutive host defense molecules, induction of proinflammatory cytokines leads to the production of additional anti-bacterial factors such as hBD-2 [39]. Inflammation in the aqueous can result in the formation of a hypopyon, which is an intense neutrophil accumulation that collects on the bottom of the anterior chamber against the cornea. Also occurring during inflammation is the formation of fibrin clots that can trap particles providing some protection against infecting bacteria, but in addition can lead to scarring of tissues surrounding the anterior or posterior chamber. With time, the clots can be dissolved by the action of urokinase, which is available in a relatively large supply [155]. 

The vitreous humor also contains a variety of anti-bacterial factors and in response to bacteria produces cytokines and chemokines that boost the host defenses. An unidentified protein in bovine vitreous humor can kill *S. aureus*, but the addition of a protease inhibitor allows bacterial survival and replication [156]. Complement is present and can contribute to the host defense; guinea pigs treated with cobra venom factor, to create a complement deficient condition, are more susceptible to infection than untreated control animals [157,158]. Also available for host defense are lysozyme, hBD-1, and bactericidal-permeability increasing protein (BPI) [154,159,160]. 

The cells surrounding the vitreous cavity, especially those associated with the retina, can respond to bacteria in an extensive fashion. Among the immune-related molecules produced are: IL-1β, IL-6, CXCL1, CXCL2, SLPI, INFγ, MIP2, IL-17A, cathelicidin-related peptide (CRAMP), hBD-1, and hBD-2 [159,161,162,163,164,165,166,167]. Up-regulated as part of the host defense are E-selectin and ICAM-1, an upregulation induced by the presence of peptidoglycan and teichoic acid of *S. aureus* [168]. The induction of this varied host response has been ascribed to the interaction of bacterial components with the TLR2 receptor [164,169,170]. Also active in the host response is the NOD-1 receptor that recognizes cell wall peptidoglycan [167]. The microglia cells from the retina when grown in cell culture were found to respond to *S. aureus* or to its peptidoglycan or lipoteichoic acid [170]. The response of the microglia cells was mediated by TLR2 and the addition of a ligand binding to this receptor caused an increase in the amount of TLR2 on the cell surface resulting in a decrease in the inflammatory products and an increase in the phagocytic function of these cells. 

Intense inflammation is harmful to ocular tissues and the response to bacterial infection needs to be limited. The retina is protected by the FasL ligand system, which is capable of inducing apoptosis in cells (e.g., neutrophils) entering the vitreous [171]. Mice lacking the FasL system undergo a far more severe infection with *S. aureus* than wild type mice; the severity of an infection with 500 CFU in the knockout mouse is equivalent to the infection initiated by 5000 CFU in a wild type mouse [172]. Injection of viable *S. aureus* into the vitreous resulted in bacterial growth from 10^2^ CFU to 10^6^ CFU in 24 h with such high numbers of bacteria remaining for 72 h [126]. This infection eliminated retina function within 12 h. 

In addition to non-specific host defenses, the tissues surrounding the vitreous can benefit also from specific immune reactions, especially those mediated by antibody. Human immunoglobulin injected into the rabbit vitreous remained in the vitreous for 5 days [173]. Normal pooled immunoglobulin was found to contain antibody to at least two *S. aureus* toxins (beta-toxin and toxic shock syndrome toxin 1 [TSST-1]) and was protective against the injection of concentrated *S. aureus* culture supernatant. Such protection was present when the immunoglobulin was injected with the culture supernatant or when the immunoglobulin injection was delayed for up to 6 h [173,174]. Specific antibody was also shown to be protective in the vitreous. The injection of PVL caused extensive inflammation, but humanized antibody to PVL protected the eye from this toxin [175]. The active humoral response to *S. aureus* present in the rat vitreous resulted in production of IgM antibody specific for the teichoic acid [176]. The IgM titer peaked by day 21 and started to decline after day 30. In rabbits, the injection of *S. aureus* into the vitreous resulted in bacterial growth from day 3 to 14 followed by a decline in the CFU until day 30 when no CFU remained [177]. IgG antibody to teichoic acid appeared in the serum, tears, aqueous humor, and vitreous humor with the serum having the highest titer until day 14. At day 14 and onward the highest titer of the IgG antibody was found in the vitreous humor. IgA antibody to teichoic acid was present in tears and vitreous humor on days 14, 21, and 30. The most important aspect of the antibody response was that the IgG titer in the vitreous was inversely proportional to the CFU in the vitreous showing how such antibody is protective against *S. aureus* [177]. 

The host defense systems protecting the vitreous are subject to disruption as is seen in infections of animals with a genetic knockout of one or more host defenses. Those knockouts that limit inflammation demonstrate the importance of inflammation in limiting the bacterial load in the vitreous. The injection of an antibody specific for neutrophils created a condition in which a *S. aureus* infection of the vitreous achieved only a relatively low clinical score, but allowed the bacterial load to become significantly higher than the controls lacking the antibody [178]. Another means of compromising the defenses of the vitreous is to induce a diabetic state in the animal. Diabetic mice are prone to developing endogenous endophthalmitis; that is, bacteria injected intravenously can penetrate the barrier between the blood vessels and the vitreous humor. Mice not rendered diabetic demonstrated the movement of bacteria from the blood to vitreous in only 7% of the cases whereas mice rendered diabetic for 3 months experienced bacterial movement from the blood to the vitreous in 58% of the animals [179,180]. 

### 6.4. Conclusions

The trauma of surgery or of an intravitreal injection creates conditions prone to the passage of bacteria into the inner eye. The inner eye is defended by a spectrum of host defense factors as evidenced by the ability of aqueous humor to kill bacteria. The standing defense against infection is augmented by the intense response inclusive of a potent inflammatory process. Experts have shown that specific immunity afforded by the injection of pooled immunoglobulins can potentially prevent or treat bacterial endophthalmitis. Given the growing problem of antibiotic resistance, passive immunization could become a common tool in dealing with *S. aureus* endophthalmitis. 

## 7. Final Comment

The eyelid, tear duct, and conjunctiva are in contact with the tear film that contains multiple soluble factors able to protect against bacterial infection, but *S. aureus* infections of these sites are commonly encountered among the general population. Such infections are not sight-threatening unless the cornea becomes involved. Corneal infections can be challenging because the combination of the immune response and the action of bacterial toxins can cause considerable tissue damage resulting in scarring that reduces visual acuity. Likewise, infections of the inner eye involve a potent host response that together with bacterial toxins can damage tissues critical to vision, especially the retina. 

## Figures and Tables

**Table 1:** Antibacterial Proteins of the Tear Film Aqueous Layer.

Aqueous Layer Protein	Activity of the Protein
Lysozyme	Comprises 20–30% of the protein; enzymatically cleaves cell wall peptidoglycan killing the bacteria, but not that of *S. aureus* [35]
Lactoferrin	Comprises 20–30% of the protein; binds iron, limiting bacterial growth and its cationic detergent effect can lyse bacteria [35,46]
Lipochalin	Binds iron, limiting bacterial growth [47]
Complement	Complement system in tears is functionally active [48]
Secretory phospholipase A2	Lipolytic enzyme activity on phospholipids of bacterial membranes is lethal for gram-positive bacteria; a major defense against *S. aureus* [49]
Secretory leukocyte protease inhibitor (SLPI)	Cationic charge can bind bacterial membranes killing the organism and can inhibit some bacterial proteases [50]
Surfactants A and D	Bind and aggregate bacteria favoring their phagocytosis [51,52]
Glycoprotein 340	Binds bacteria favoring their phagocytosis [53]
Antimicrobial peptides	Have bactericidal effects [54]

**Table 2:** Factors Active in *S. aureus* Keratitis *.

Eye Protections to Be Avoided by Bacteria		Bacterial Binding Proteins	Epithelial Cell Response	Inflammation and Tissue Damage Mediators
Tear Film	Mucous Layer			
PLA2 Mucins Surfactant Protein Immunoglobulin Leukocytes Complement Cytokines AMP’s SLPI Lactoferrin Lipochalin	Mucins Leukocytes Immunoglobulins Cytokines	Fibronectin Binding Protein Collagen Binding Protein	IL-1α IL-1β IL-6 IL-8 IL-17 TNF-α LL-37	Teichoic Acid Alpha-Toxin Gamma-Toxin PVL ** SSL ** S. aureus elastase **

* Abbreviations: PLA2—phospholipase A2; AMP’s—anti-microbial peptides; SLPI—secretory leukocyte protease inhibitor; PVL—Panton-Valentine Leukocidin; and SSL—super-antigen like protein. ** Findings on PVL, SSL, and *S. aureus* elastase need further analysis to better understand their role in virulence.
